# Oil-in-Water Emulsions Stabilized by Ultrasonic Degraded Polysaccharide Complex

**DOI:** 10.3390/molecules24061097

**Published:** 2019-03-20

**Authors:** Yujie Li, Dong Xiang, Bo Wang, Xiaoyue Gong

**Affiliations:** 1 College of Food Science, Hainan University, No.58 Haikou 570228, China; lyj20162012@126.com (Y.L.); 15607662867@163.com (B.W.); gxylay120408@163.com (X.G.); 2 Engineering Research Center of Utilization of Tropical Polysaccharide Resources, Ministry of Education, No.58 Haikou 570228, China

**Keywords:** ultrasound, propylene glycol alginate, xanthan gum, emulsion stability

## Abstract

The effects of ultrasound on the molecular weight distribution and emulsifying properties of both xanthan gum (XG) and propylene glycol alginate (PGA) were investigated. The results showed that ultrasonic treatment at different intensities decreased the apparent viscosity and narrowed the molecular weight distribution. Higher intensity increased the effectivity of the sonochemical effect. Ultrasound degradation did not change the primary structure of the PGA-XG complex, and SEM analysis showed that the morphology of the original polysaccharide differed from that of the degraded polysaccharide fractions. The ultrasonic intensities and treatment times had a substantial influence on the stability of the polysaccharide-stabilized oil-in-water (O/W) emulsions. The O/W emulsion stabilized by the polysaccharide treated with 270 W ultrasound waves for 7 min led to the smallest average particle size (detected via fluorescence microscopy) and showed stability against aggregation in O/W emulsions.

## 1. Introduction

Ultrasonic waves have been applied to a variety of physical and chemical processes such as emulsification [1], dispersion, homogenization, and various chemical reactions [2]. Furthermore, research has mainly focused on the novel effects of ultrasound on both the properties and functionality of products, such as the emulsification and rheological properties of food hydrocolloids [3,4]. Moreover, ultrasonic treatment is an effective and energy-saving method for preparing and processing polymer particles [5].

Xanthan gum (XG) is a type of anionic exocellular polysaccharide produced by the aerobic fermentation of sugars by the bacterium *Xanthomonas campestris*. Importantly, the rheological properties of the aqueous phase are influenced by XG, which is used as a thickening agent and plays an essential role in emulsion stability, contributing to the formation of a gel-like network [6]. Since XG is a hydrophilic polymer, it preferentially enters hydrogen bonding with water. Its surface-active properties as well as its rheological properties can be modified when it is used in combination with propylene glycol alginate (PGA) [5].

PGA is a high molecular weight linear polysaccharide with 50–85% esterified carboxyl groups that is derived from the reaction between propylene oxide and alginic acid [7,8,9] Additionally, PGA is composed of 31–65% 1,4-linked-d-mannuronic acid and 69–35% l-guluronic acid and can be utilized as a stabilizer and foaming agent [10,11].

The degradation of naturally occurring biopolymers has become a key goal for colloid studies, due to the necessity of the reduction in molecular weight or particle size, or confinement within a narrow range size distribution [12,13], to meet application requirements [14]. Recently, several techniques for degradation have been reported, of which ultrasonic degradation was considered to be the optimum method for the control of the molecular weight of degraded products. In addition, in response to ultrasonic treatment, degraded polymers are transformed into simple chemical structures, whereas other methods may promote alterations and thus the behavior of materials [10].

In this study, the emulsifying properties of PGA-XG complexes were characterized in response to treatment with various intensity levels and durations. The effects of ultrasonic treatment on the molecular weight and apparent viscosity of the polysaccharide solution at two intensities were investigated as a function of time. The preliminary characterizations of the polysaccharides before and after ultrasonic treatment were evaluated. Furthermore, the influences of ultrasonic intensity and treatment time on the stability of the emulsions (droplet growth and gravitational separation) were determined. In addition, morphological characteristics of oil-in-water (O/W) emulsions were examined with fluorescence microscopy.

## 2. Results and Discussion

### 2.1. Viscosity Measurement

**Figure 1:**
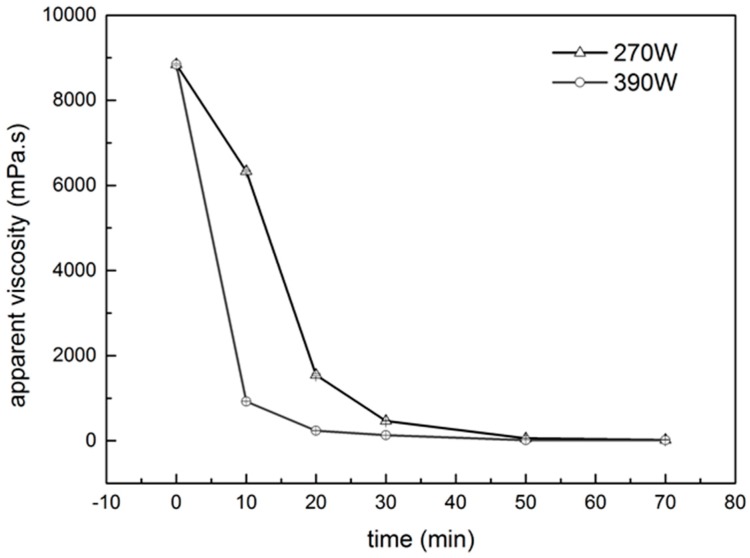
Effect of ultrasonic intensities and treatment time on the apparent viscosity of PGA-XG complexes (0.06 g/L; PGA:XG 3:7 wt, viscosity measured at 30 °C). Data are expressed as mean ± SD for three independent replicates (*n* = 3) of each sample.

The apparent viscosities of the PGA-XG complexes under ultrasonic treatment at different intensities are shown in Figure 1 as a function of time. For both intensities, the apparent viscosity of the polysaccharide solution decreased with time. At higher power (390 W), the apparent viscosity decreased steeply from the initial value to a minimal level (similar to that of water) within 20 min of exposure to ultrasound. At lower power (270 W), the apparent viscosity decreased to a minimal level (similar to that of water) within 30 min of ultrasound treatment. At the beginning of treatment (after 10 min) at 390 W, the apparent viscosity decreased rapidly from 8700 mPa/s to 300 mPa/s. As the treatment time increased from 30 to 70 min, a decrease in the rate of the smaller change in apparent viscosity was observed and reached an optimum value (15 mPa/s) after 70 min. In comparison, the reduction of apparent viscosity was higher and considerably faster at high ultrasonic intensity. A stronger reduction in apparent viscosity was obtained using a higher intensity, implying that ultrasonic intensity has a significant influence on the ultrasonic degradation of polysaccharides. This degradation may be due to cavitation action (mechanical effect), which increases with higher ultrasonic intensity. Subsequently, this decreased the threshold of cavitation and increased the number of cavitation bubbles [15].

### 2.2. Effect of Ultrasound on Molecular Weight Distribution

**Figure 2:**
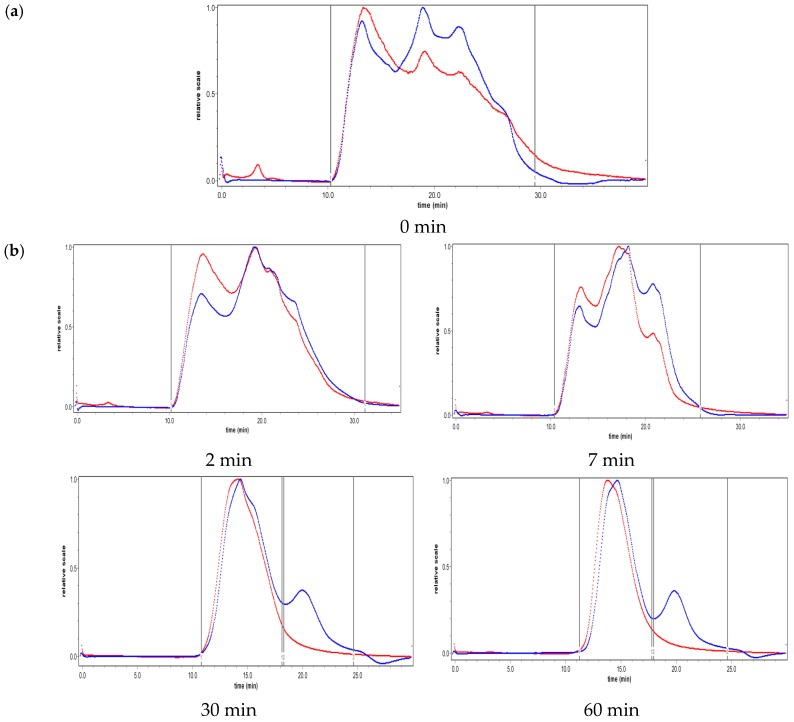

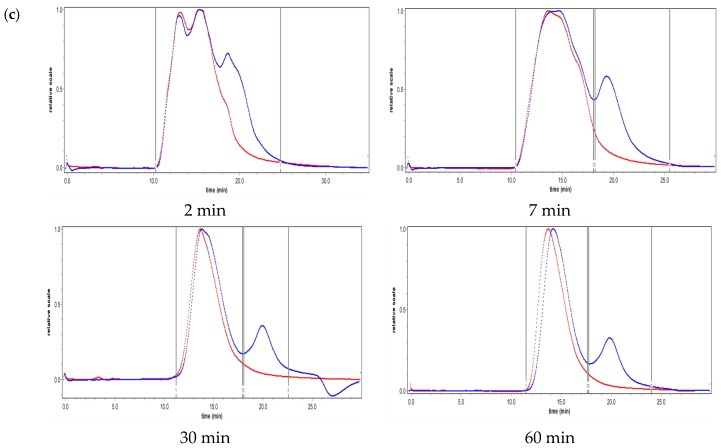
Effect of ultrasonic treatment on molecular weight distribution: (**a**) before ultrasonic treatment, (**b**) 270 W ultrasonic treatment, and (**c**) 390 W ultrasonic treatment. The red line represents the result of the light scattering detector, and the blue line represents the result of a differential refractive index detector.

In this study, GPC was employed to investigate the molecular weight distribution of the PGA-XG complex before and after ultrasonic treatment at different intensities and treatment times. The data obtained are presented in Figure 2. The molecular weight distribution of the PGA-XG complex narrowed rapidly within the first 30 min but slowed down during the remaining 30–60 min of ultrasonic treatment at 270 W (Figure 2a). The same rapid narrowing occurred but within the first 15 min and slowed down during the remaining 15–60 min of ultrasonic treatment at 390 W (Figure 2b). After ultrasonic treatment, the molecular weight distribution curve exhibited a bimodal pattern and did not change over time. Polysaccharide fracturing was mainly caused by the shearing force produced by the rapid collapse of cavitation bubbles [2,16]. Large polymers with longer chains are preferentially broken by the shearing force, whereas smaller molecules are more resilient [17]. Thus, the molecular weight distribution of the sample decreased at an early stage due to this fracturing, which was why the rate slowed as time progressed. The two kinds of polysaccharide were eventually degraded into different length short chains, so the molecular weight distribution curve became a double peak.

Ultrasonic intensity is an important factor affecting the distinct stages of acoustic cavitation: nucleation, bubble growth, and collapse [9]. A significant increase in degradation efficiency was observed with increasing ultrasonic intensity. It can be seen from the Figure 2 that at the same time point the molecular weight distribution curve of the polysaccharide degraded by 390 W is narrower than the molecular weight distribution curve of the polysaccharide degraded by 270 W ultrasound.

### 2.3. Effect of Ultrasound on the Primary Structure of Polysaccharides

Native and ultrasonically-treated polysaccharides, including PGA and XG (treated at ultrasonic intensities of 270 W and 390 W, for 60 min), were selected for further characterization of their primary structure.

**Figure 3:**
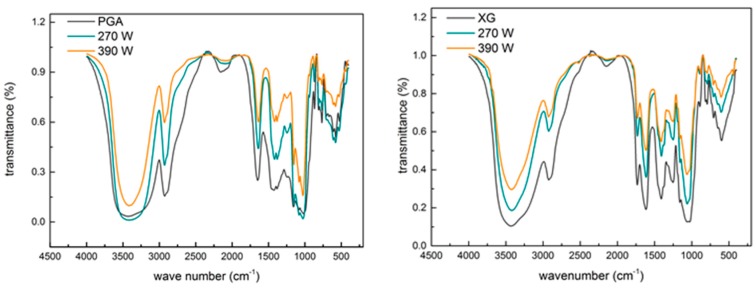
Fourier transform infrared (FTIR) spectra of native and 270 W and 390 W ultrasonically treated propylene glycol alginate (PGA) and xanthan gum (XG).

Figure 3 shows the FTIR spectra of native and ultrasonically treated PGA and XG, all of which had virtually identical characteristic absorption peaks. All samples showed a wide and strong absorption peak around 3429 cm^−1^, which was assigned as -OH. The stretching and bending vibration absorption peak at around 2924 cm^−1^ corresponded to C-H [18]. The peak at 1736 cm^−1^ could be attributed to the stretching C=O in the acetyl groups [19]. The intense peak at 1640 cm^−1^ can be attributed to intramolecular hydrogen bonds. The strong extensive absorption peaks at 1000–1200 cm^−1^ suggest the presence of C-O-H, C-C, and C-O-C [2,3,15], and the absorbance bands around 874 cm^−1^ and 807 cm^−1^ are characteristic of mannose in PGA and XG. The high similarity in the FTIR spectra between ultrasonically-treated PGA or XG and their corresponding native forms suggest that both the repeating units and the primary structures of PGA and XG were not affected by the ultrasonic treatment.

**Figure 4:**
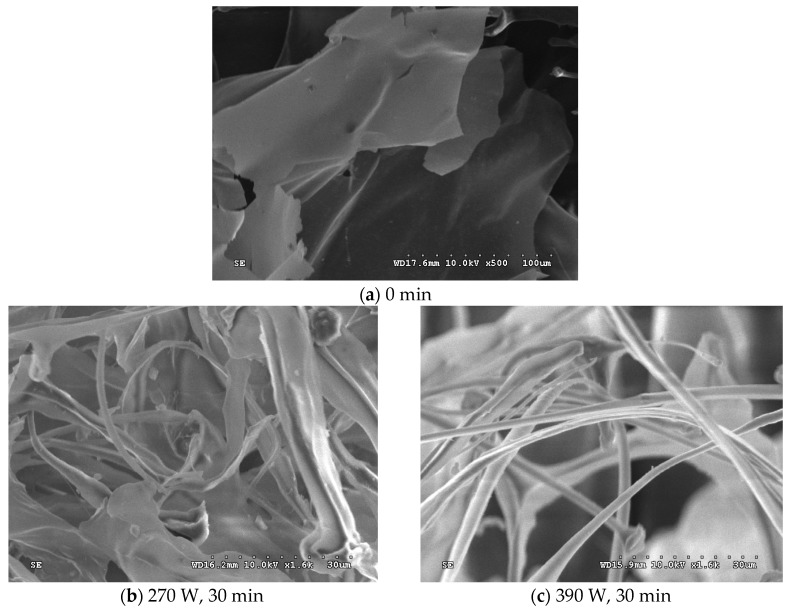
Scanning electron microscopy (SEM) images of polysaccharides under ultrasonic treatment: (**a**) Native polysaccharide; (**b**) 270 W ultrasonic treatment for 30 min; and (**c**) 390 W ultrasonic treatment for 30 min.

SEM was employed as an effective tool to qualitatively analyze the changes in surface morphology of native polysaccharides (PGA and XG) and ultrasonically-treated polysaccharide fractions, and the results are presented in Figure 4. The surfaces of the native polysaccharides and the degraded polysaccharides showed clear variations in size and shape. The native polysaccharides (Figure 4) showed rough surface-like clouds or a flake-like morphology. In contrast, after ultrasonic treatment, the polysaccharide fractions (Figure 4) showed a rough appearance with extensive pores detected on the surface, or a mesh-like morphology. Moreover, as the ultrasonic intensity increased from 270 to 390 W, the microstructure was dramatically fragmented and small segments or pores were obtained. The network interconnections or aggregates were thoroughly split into thin and fragmented branches, generated from fewer chain strands or looser structures. These results could be attributed to the intensive cavitation, turbulent shear, and instantaneous high-pressure drop involved in the ultrasound treatment process. With increasing ultrasonic intensity and treatment time, the ultrasonic energy was sufficient to break down strong bonds, such as the glycosidic linkages that connect the sugar units.

### 2.4. Effect of Ultrasound on Interfacial Tension

**Figure 5:**
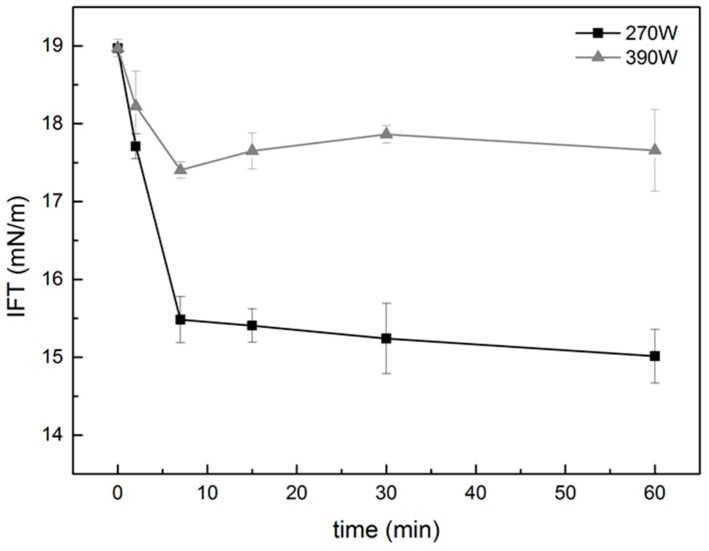
Effect of different ultrasonic intensities and treatment times on the interfacial tension measured at the coconut oil–polysaccharide solution interface. Data are expressed as mean ± SD of three independent replicates (*n* = 3) of each sample.

The interfacial characteristics of surface-active ingredients are a significant factor in determining their ability to form and stabilize emulsions [16]. This study examined the interfacial tension between polysaccharide solutions and coconut oil (Figure 5). The experimental results indicated that ultrasonic power reduces the interfacial tension at the oil-water interface, thus indicating that ultrasonic treatment can change the adsorption characteristics of the PGA-XG complexes at this interface (Figure 5). However, different ultrasonic intensities and treatment times showed variations in their ability to decrease interfacial tension at the O/W interface. We found that PGA-XG complexes significantly reduced the interfacial tension compared to water (23.5 mN/m) at coconut oil–polysaccharide solution interfaces. The presence of both hydrophilic and hydrophobic bonds enabled the effective adsorption of the PGA-XG mixture at the interface of the coconut oil. According to Figure 5, during the early stages of ultrasonic treatment, the interfacial tension between the oil and polysaccharide solution decreased rapidly, which was consistent with the results of apparent viscosity. Furthermore, the amphiphilic polysaccharides generated thick interfacial layers after they were adsorbed onto the oil droplet surface. The cavitation of the ultrasonic power degrades polysaccharides into smaller fractions [4,20], which can be adsorbed quicker to the oil droplet surface, where they form a layer [21], which may contribute to the decrease of interfacial tension.

### 2.5. Particle Size and Size Distribution of Fresh Emulsions

**Figure 6:**
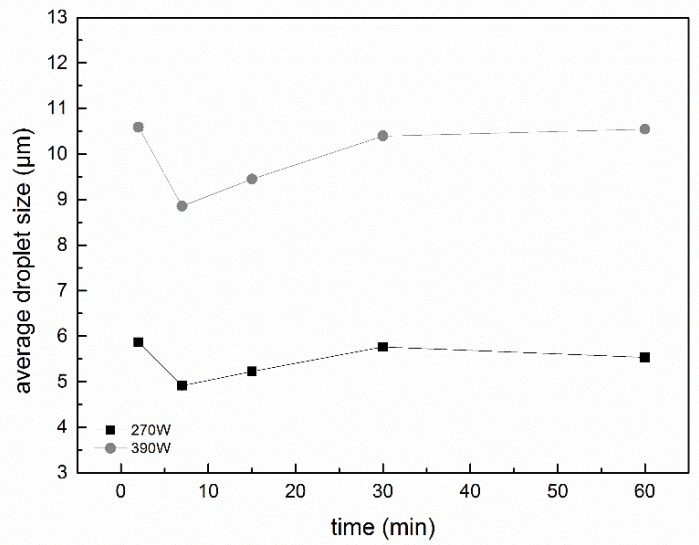
Average droplet size of 5% coconut oil O/W emulsions prepared by different ultrasonic intensity and treatment times. Data are expressed as mean ± SD for three independent replicates (*n* = 3) of each sample.

**Figure 7:**
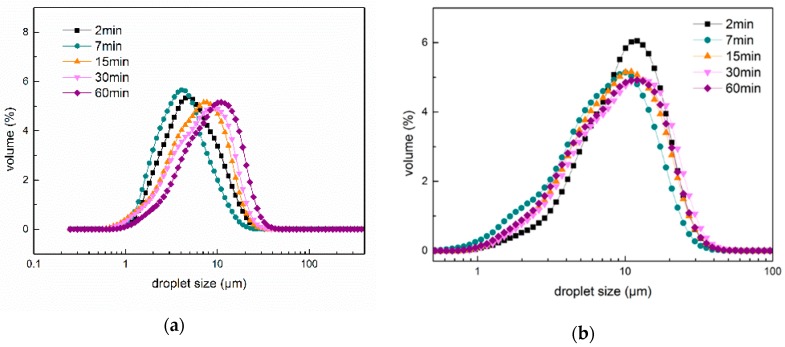
Droplet size distribution of 5% coconut oil O/W emulsions stabilized by different ultrasonic intensities and treatment times: (**a**) Droplet size distribution of O/W emulsions prepared by 270 W ultrasonic power and (**b**) droplet size distribution of O/W emulsions prepared by 390 W.

Ultrasonic intensity and treatment times can greatly influence O/W emulsion stability; therefore, appropriate parameters must be found to optimize the shelf life of emulsions. The average droplet size and distributions of 5% coconut oil O/W emulsions stabilized by PGA-XG complexes and prepared by different ultrasonic intensities and treatment times were tested. The droplet size distribution and average particle size of coconut oil O/W emulsions are shown in Figure 6 and Figure 7. The shear rate of ultrasonic power decreased the droplet size of the coconut oil in the O/W emulsion [22]. The mixture of PGA and XG formed a film around the droplet surface, which better maintained the stability of the 5% coconut oil O/W emulsions [23]. The polysaccharides formed a relatively thick hydrophilic coating around the coconut oil droplets and generated a strong steric repulsion that prevented aggregation in the O/W emulsions. As shown in Figure 6, the average particle size of the O/W emulsions prepared by a 270 W intensity ultrasound wave was significantly smaller than those prepared by 390 W. The experimental results showed that the ultrasonic time greatly influenced the average particle size of the emulsions. The O/W emulsion prepared by 270 W for 7 min exhibited the smallest average particle size.

As shown in Figure 7, the emulsions prepared by 270 W ultrasound for 7 min and 2 min presented the narrowest droplet size distribution and resulted in the best stability. However, the droplet size distribution of the emulsions widened after prolonged ultrasonic treatment. This may be related to the reduction in emulsion viscosity, causing partial aggregation. However, all droplet size distributions of the coconut oil O/W emulsions prepared by 390 W ultrasonic waves were wider than that prepared by 270 W ultrasonic waves. This may be because the high-intensity ultrasonic effect can degrade the polysaccharide chain into shorter chains; therefore, the effect of the polysaccharide chain in stabilizing the O/W emulsions is worse.

### 2.6. Microstructure of the Emulsions

**Figure 8:**
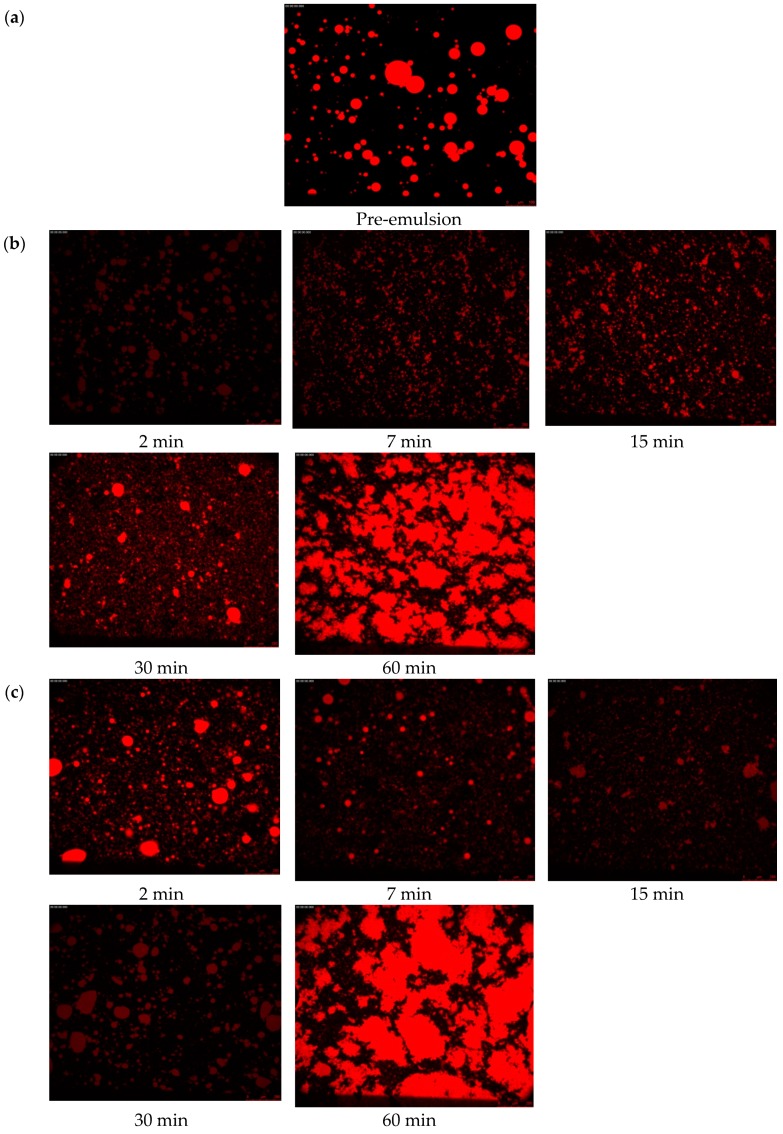
Microstructures of fresh coconut (O/W) emulsions treated by ultrasonic waves and observed via fluorescence microscopy: (**a**) pretreatment emulsion, (**b**) O/W emulsions treated by 270 W, and (**c**) O/W emulsions treated by 390 W.

The fluorescence microscopic images of the 5% coconut O/W emulsions stabilized by PGA-XG complexes prepared by different ultrasonic intensities and treatment times are presented in Figure 8. Compared to the image of pre-emulsion (Figure 8a), the droplet sizes of PGA-XG (3:7, wt) stabilized emulsions were slightly smaller after ultrasonic treatment and showed insignificant variation when the ultrasonic exposure was extended. Likely, the ultrasonic waves decreased the droplet size of the internal phase due to cavitation [24]. However, with prolonged time the phenomenon of droplet aggregation occurred in the O/W emulsions, which may be related to the low apparent viscosity of the O/W emulsions (Figure 8c). As shown in Figure 8b, the O/W emulsions prepared by 270 W intensity for 7 min and 15 min presented a small droplet size.

### 2.7. Visual Phase Separation

**Figure 9:**
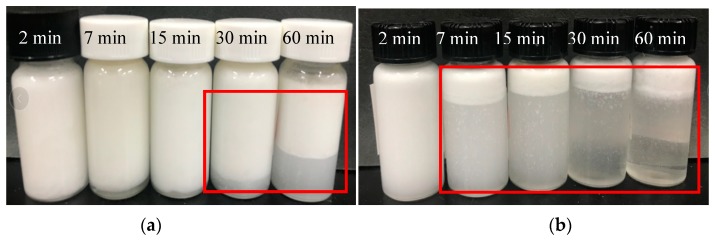
General appearance of 5% coconut oil O/W emulsions prepared by different ultrasonic intensities and treatment times after 30 days of storage at 30 °C: (**a**) 270 W and (**b**) 390 W.

The appearance of the emulsions after storage at 30 °C for a period of time is shown in Figure 9. After 30 days, partial phase separation was observed. The O/W emulsions prepared by 270 W for both 2 and 7 min were stable against gravitation separation. The majority of those prepared by 390 W showed phase separation, which is consistent with the results of the average droplet size (Figure 9, after 30 days of storage). High-intensity and long-term ultrasonic treatment degraded the polysaccharide into short chains, which have a poor ability to stabilize O/W emulsions. Therefore, phase separation occurred in the O/W emulsions during storage.

## 3. Materials and Methods

### 3.1. Materials and Polysaccharide Solution Preparation

PGA (75% degree of esterification) was purchased from Yuanye Bio-Technology Co., Ltd. (Shanghai, China). Xanthan gum was purchased from Sigma-Aldrich (St. Louis, MO, USA), and commercial coconut oil was purchased from a local supermarket (QianCheng food company, Hainan, China). Nile Red was purchased from Yuanye Bio-Technology Co., Ltd. (Shanghai, China). All other chemicals utilized were of analytical grade and were purchased from Sinopharm Chemical Reagent Co., Ltd. (Shanghai, China). Purified water was prepared using a Barnstead E-pure system (Dubuque, IA, USA).

The polysaccharide solution was prepared as follows: 0.18 g of PGA and 0.42 g XG were dispersed in 100 mL pure water and gently stirred at 30 °C. The dispersion was incubated overnight to ensure complete hydration of the complex.

### 3.2. Viscosity Measurement

Apparent viscosity measurements were carried out using a Brookfield viscometer (DV3TLVTJ0, Middleboro, MA, USA) equipped with a spindle numbered LV-1 or LV-2 at 50 r/min. The sample cell was filled with the polysaccharide solution. The temperature was maintained at 30 °C by circulating water from a constant temperature circulator.

### 3.3. Preparation of O/W Emulsions

Coconut oil (5 g) was added to the 95 g as-prepared polysaccharide solution (0.6% wt). The mixture was then pre-homogenized with a high shear homogenizer (FJ-200; Biaoben Instruments, Shanghai, China) at a speed of 18,000 rpm for 2 min at 30 °C. The resulting coarse emulsion was treated with ultrasound waves (FS-600N, Shangchao, Shanghai, China) at a constant power of 270 W or 390 W for 2 min, 7 min, 15 min, 30 min, and 60 min. The coarse emulsion was placed into a 200 mL glass containing cold water to prevent overheating of the emulsion during ultrasound treatment. Finally, 0.005 g sodium azide (NaN_3_) was added as an antimicrobial preservative.

### 3.4. Determination of Average Molecular Weight and Molecular Weight Distribution

The molecular weight distribution was determined by gel permeation chromatography (GPC) following the method described by Houben [25] with minor modifications. 20 μL of the sample solution was injected and eluted with 0.02% NaN_3_ at 40 °C at a flow rate of 0.5 mL/min. The eluent was monitored with both a Waters 515 laser light scattering detector (Water, MA, USA) and a differential refractive index detector (Water, MA, USA).

### 3.5. Fourier Transform Infrared (FTIR) Spectroscopic Analysis

The primary structure of the freeze-dried PGA-XG complexes before and after ultrasonic treatment was investigated using a FTIR spectrometer (Bruker Optics Co., Berlin, Germany) at a wavenumber range of 500–4000 cm^−1^ using potassium bromide (KBr) pellets.

### 3.6. Interfacial Characteristics Measurement

The interfacial tension between the oil and water was measured by a drop-shape analysis instrument (DropMeter A-60, MAIST, Ningbo, China) at 30 °C, according to a previously reported method [11]. The screw control above the injector was twisted to form a suitable oil droplet size and to measure the falling droplet. The droplets were allowed to stand in the coconut oil for 5 min.

### 3.7. Scanning Electron Microscopy (SEM)

The freeze-dried polysaccharide samples were fixed onto a copper stub. After sputtering with a layer of gold, SEM images were observed and recorded using a SEM (Cannon Co., Tokyo, Japan) under high vacuum conditions at accelerating voltage.

### 3.8. Measurement of Droplet Size

The average droplet size and distribution of coconut oil O/W emulsions were determined by a laser particle size analyzer (Ineas physical optics instrument, Co., Ltd., WJ-60, Shanghai, China). To measure both the average particle size and particle size distribution, 0.5 mL of the sample was added to the sample cell. The experiment was conducted at 30 °C. The average particle size and the particle size distribution curve were obtained with the software WJ-60 that came with the instrument.

### 3.9. Fluorescence Microscopy

A fluorescence microscope (Leica DMI DM6000B, Leica Microsystems, Heidelberg, Germany) was used to observe the microstructure of the O/W emulsions. This microscope operated in fluorescence mode using a 10× objective with numerical aperture of 0.40. The oil droplets were stained with Nile Red fluorescent dye. An aliquot of emulsion sample was placed onto a microscope slide. A cover slip was placed on top of the microscope slide, ensuring that no air bubbles were trapped inside. The samples were observed at 30 °C.

### 3.10. Visual Observation of the Phase Separation

The O/W emulsions (10 mL) were transferred to transparent glass test tubes, sealed with plastic caps, and stored at 30 °C for 30 days. Physical phase separation was monitored.

### 3.11. Statistical Analysis

All measurements were performed in triplicate, and statistical analysis was performed using the Statistical Analysis Systems (Origin 9.0, Originlab, Studio, CA, USA) software package.

## 4. Conclusions

In conclusion, the primary characterization, molecular weight, and effect of ultrasonic treatment on the stability of O/W emulsions of PGA-XG complexes were investigated. The results obtained show that the complexes had a large reduction in apparent viscosity. The preliminary structure of the PGA-XG complex before and after ultrasonic treatment did not change. According to SEM analysis, the morphology of the original complexes differed from the degraded polysaccharide fractions and exhibited variations in the maintenance of the stability of the O/W emulsions. At a specific ultrasonic power, small and stable oil droplets formed in the polysaccharide mixture, which prevented droplet aggregation due to the formation of a dense film at the interface. Different ultrasonic intensities and treatment times affected the degradation of polysaccharides. The O/W emulsion treated at 270 W for 7 min exhibited the best stability. This may be related to the molecular weight distribution of polysaccharide fractions, which is uniform under high-power long-term treatment, and the apparent viscosities of the O/W emulsions being too low to maintain stability. The O/W emulsion subjected to short-time, low-power treatment achieved high apparent viscosity and resisted aggregation. However, treatment with low ultrasonic power was insufficient to break the oil droplets into particles with a small diameter. Therefore, ultrasonic power is a useful means for the degradation of polysaccharide and O/W emulsion preparations. In subsequent studies, the effect of ultrasonic treatment on the rheological properties of PGA and XG mixtures will be further studied.
